# The *Caenorhabditis elegans* Werner Syndrome Protein Functions Upstream of ATR and ATM in Response to DNA Replication Inhibition and Double-Strand DNA Breaks

**DOI:** 10.1371/journal.pgen.1000801

**Published:** 2010-01-08

**Authors:** Se-Jin Lee, Anton Gartner, Moonjung Hyun, Byungchan Ahn, Hyeon-Sook Koo

**Affiliations:** 1 Department of Biochemistry, College of Life Science and Biotechnology, Yonsei University, Seoul, Korea; 2 Wellcome Trust Centre for Gene Regulation and Expression, School of Life Sciences, The University of Dundee, Dundee, United Kingdom; 3 Department of Life Sciences, University of Ulsan, Ulsan, Korea; The University of North Carolina at Chapel Hill, United States of America

## Abstract

WRN-1 is the *Caenorhabditis elegans* homolog of the human Werner syndrome protein, a RecQ helicase, mutations of which are associated with premature aging and increased genome instability. Relatively little is known as to how WRN-1 functions in DNA repair and DNA damage signaling. Here, we take advantage of the genetic and cytological approaches in *C. elegans* to dissect the epistatic relationship of WRN-1 in various DNA damage checkpoint pathways. We found that WRN-1 is required for CHK1 phosphorylation induced by DNA replication inhibition, but not by UV radiation. Furthermore, WRN-1 influences the RPA-1 focus formation, suggesting that WRN-1 functions in the same step or upstream of RPA-1 in the DNA replication checkpoint pathway. In response to ionizing radiation, RPA-1 focus formation and nuclear localization of ATM depend on WRN-1 and MRE-11. We conclude that *C. elegans* WRN-1 participates in the initial stages of checkpoint activation induced by DNA replication inhibition and ionizing radiation. These functions of WRN-1 in upstream DNA damage signaling are likely to be conserved, but might be cryptic in human systems due to functional redundancy.

## Author Summary

Werner syndrome is a premature aging syndrome associated with genomic instability. The protein linked to Werner syndrome, WRN, has both helicase and exonuclease activities and is thought to be involved in DNA repair, including the resolution of replication fork arrest as well as in telomere maintenance. However, no definite and detailed role of the protein has been elucidated in vivo. We take advantage of the *Caenorhabditis elegans* germ cell system to explore DNA damage response defects associated with WRN, and we focus particularly on the role of *wrn* in the cell cycle checkpoint in response to DNA replication blockage and ionizing radiation (IR). We show that WRN functions together with RPA upstream of *C. elegans* ATR in the intra S-phase checkpoint pathway, and upstream of *C. elegans* ATM and RPA in the cell cycle arrest pathway triggered by IR–induced double-strand DNA breaks. These functions of WRN in upstream DNA damage signaling are likely to be conserved, but not obvious in human systems due to functional redundancy.

## Introduction

Werner syndrome (WS) is associated with rapid acceleration of aging, and is caused by mutations in the RecQ family DNA helicase gene, *WRN* [1]. Clinical symptoms of WS include short stature, hair-graying, cataract formation, type II diabetes, osteoporosis, atherosclerosis, and neoplasm of mesenchymal origins [2]–[5]. The role of WRN in premature aging may be linked to telomere regulation. WS fibroblasts have a reduced replicative life span, which is alleviated by the forced expression of the human telomerase (hTERT) gene [6]. A link between WRN, telomere DNA metabolism and progeria has been demonstrated in mouse *Wrn* and *Terc* (telomerase RNA) knockout models [7]. Symptoms of Werner syndrome appeared in late-generation *Wrn* and *Terc* double mutant mice together with accelerated telomere loss, while the corresponding single mutants did not show such phenotypes. The role of WRN in telomere DNA metabolism is also supported by a report showing that WRN localizes to telomeric DNA during the S-phase of telomerase-defective ALT cells and by its ability to resolve telomeric D loops via its helicase and nuclease functions *in vitro* [8].

WRN is one of the five human RecQ family helicases and possesses an N-terminal exonuclease domain homologous to *E. coli* RNaseD [9]–[12]. The two enzymatic activities of WRN and the presence of chromosomal rearrangements and deletions in WS cells led to the notion that WRN is involved in the resolution of stalled replication forks, and in various DNA repair and recombination pathways [2]–[5]. This view is supported by the fact that WRN interacts with proteins known to participate in DNA processing, such as FEN1, RPA, polymerase δ, PCNA, poly(ADP-ribose) polymerase 1, NBS1, γ-H2AX, and Ku80/70 [3],[4],[13],[14]. Moreover, WRN is a 3′—5′ helicase capable of unwinding a variety of DNA structures that act as intermediates in recombinational repair of stalled replication forks such as Holliday junctions, bubble substrates, D-loops, flap duplexes, and 3′-tailed duplex substrates [2],[3].

Nevertheless, relatively little is known about the cellular functions of WRN in DNA repair and in DNA damage signalling. Furthermore, it is not known if any of the in vitro biochemical activities reported for WRN are required for its function in vivo. Human WRN was implicated in a G2 cell cycle checkpoint, in response to the inhibition of chromosomal decatenation [15]. In addition, human WRN is required for full ATM activation and for slowing down S-phase progression in response to DNA interstand crosslinks or in response to the inhibition of DNA replication [16].

**Figure 1:**
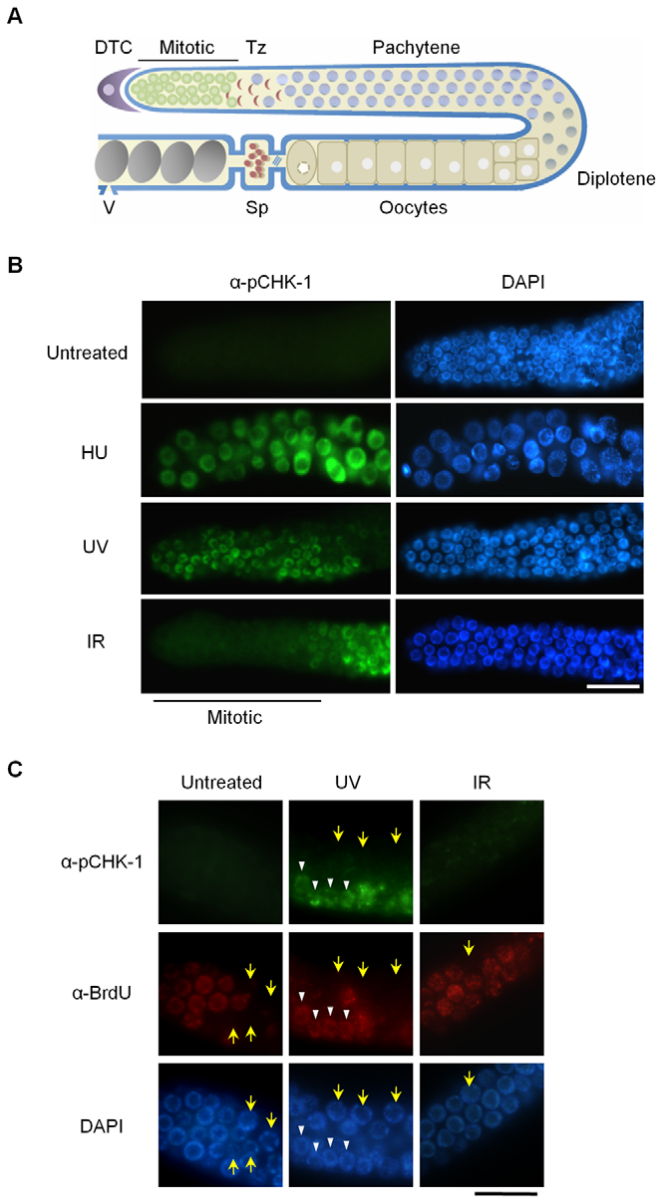
CHK-1 phosphorylation in *C. elegans* germ cells after DNA replication inhibition or DNA damage. (A) Schematic representation of a gonad arm in an adult *C. elegans* hermaphrodite. DTC, distal tip cell; Mitotic, mitotically proliferating region, Tz, transition zone; Sp, spermathecum containing sperms; V, vulva at the uterus containing embryos. (B,C) To inhibit DNA replication, wild-type N2 *C. elegans* worms were treated with 25 mM hydroxyurea (HU) from the L4 stage for 16 h. DNA damage was induced by irradiating one-day-old adult worms with UV (100 J/m^2^) or γ-rays (75 Gy), and cultivating them for 1 h. In (C), worms were fed with BrdU-labeled *E. coli* cells for 30 min before DNA damage and then with unlabeled *E. coli* cells for 1 h after the damage. Gonads were isolated and reacted with (B) phospho-CHK1(Ser345) antibody and with (C) both phospho-CHK1(Ser345) and BrdU antibodies. Mitotic (premeiotic) regions of the gonads were observed under a fluorescence microscope. Magnification bars, 25 μm.

Here we exploit the *Caenorhabditis elegans* germ line which is the only proliferative tissue in adult worms, as an experimental system to analyse the functions of WRN in DNA damage signalling. The gonad contains various germ cell types that are arranged in an ordered distal to proximal gradient of differentiation [17; Figure 1A]. The distal end of the gonad is comprised of a mitotic stem cell compartment, followed by a ‘transition zone’ where entry into meiotic prophase occurs. DNA replication failure and DNA double strand breaks lead to a prolonged cell cycle arrest of mitotic germ cells [18].

The *C. elegans* genome encodes four worm RecQ family proteins that correspond to their human orthologs. *C. elegans* WRN-1 is most closely related to human WRN, but has no exonuclease domain [19]. We previously showed that WRN-1 depletion by RNA leads to reduced lifespan. We also observed an increased incidence of diverse developmental defects whose frequency was further accentuated by γ-irradiation [19]; a phenotype likely associated with DNA repair defects occurring during development [20]. In addition, we provided evidence for an abnormal checkpoint response to DNA replication blockage.

In this study, we further explore DNA damage response defects associated with WRN-1 in the *C. elegans* germ cell system, and particularly focus on the role of *wrn-1* in the cell cycle checkpoint in response to DNA replication blockage and ionizing radiation. We show that WRN-1 functions together with RPA-1 upstream of *C. elegans* ATR in the intra S-phase checkpoint pathway, and upstream of *C. elegans* ATM and RPA to trigger cell cycle arrest in response to IR-induced double-strand DNA breaks.

## Results

### CHK-1 Phosphorylation in Proliferating Germ Cells of *C. elegans* Is Induced by DNA Replication Blockage and UV, and Is Associated with S-Phase Arrest

To probe activation of the DNA damage checkpoint in mitotic *C. elegans* germ cells and also to examine the relationship between WRN-1 and CHK-1, we used a commercially available antibody against the conserved Ser345 phosphoepitope of human CHK1 corresponding to *C. elegans* Ser344. In humans, CHK1 is phosphorylated by ATR on Ser317 (which is not conserved in *C. elegans*) and/or Ser345 when DNA replication is inhibited or upon DNA damage induced by UV, ionizing radiation, or genotoxic chemicals [21]. As shown in Figure 1B, CHK-1 phosphorylation was absent before induction of DNA damage, but became apparent in the nuclei of enlarged germ cells that result from inhibition of DNA replication by hydroxyurea (HU). It had been previously shown that treating *C. elegans* germ lines with the deoxyribonucleotide-depleting drug hydroxyurea leads to transient germ cell cycle arrest [22]. Germ cell cycle arrest results in enlarged cells and nuclei due to cessation of cell division while cellular growth continues unabated. CHK-1 phosphorylation was also observed in the majority of germ cell nuclei after UV radiation (Figure 1B). One hour after γ-irradiation (IR: ionizing radiation), phosphorylation of CHK-1 was observed in germ cell nuclei at the pachytene and transition stages but not in those of the mitotically proliferating region (Figure 1B).

To ask why phosphorylation of CHK1 Ser345 is detected in some cells of the proliferating region after UV treatment but not after IR treatment, we labeled S-phase cells with 5-bromodeoxyuridine (BrdU). Adult worms were exposed to BrdU for 30 min and irradiated with UV or IR, followed by incubation of 1 h in the absence of BrdU. Thereafter the gonads were simultaneously reacted with antibodies against BrdU and pCHK1-Ser345. Incorporation of BrdU into chromosomal DNA was observed in medium-sized germ cells of the mitotic region, but not in smaller and larger cells (marked by yellow arrows) that are probably in G1 or G2 phase, respectively (Figure 1C). Likewise, medium-sized UV-treated germ cells showed nuclear BrdU-staining, and only those cells (marked by white arrowheads in Figure 1C) were positive for pCHK1-Ser345, suggesting that CHK1-Ser345 phosphorylation is associated with S phase. One hour after IR treatment, none of the germ cells were positive for pCHK1-Ser345, although more than half of the IR-treated cells in the distal part of the germ line showed nuclear BrdU-staining. The observation that pCHK1-Ser345 was absent even in S phase IR-treated germ cells, agrees with the finding that phosphorylation of CHK1-Ser345 is not induced by double-strand DNA breaks in mammalian cells [23]. IR-treated germ cells in the mitotic region finally arrest in G2 phase [24], but 1 h after irradiation they were still in various phases of the cell cycle, which has been estimated to last 16–24 h [25].

### WRN-1 Is Required for CHK-1 Phosphorylation after DNA Replication Blockage, but Not after UV Radiation

Our previous RNA*i*-based results hinted that *wrn-1* might be involved in the S-phase DNA replication checkpoint [19]. To confirm this result and to further examine the function of *wrn-1* in the DNA replication checkpoint, we obtained *wrn-1* deletion mutants and backcrossed them six times to eliminate unlinked mutations (Figure S1A). The 196 bp deletion mutation of *wrn-1 (gk99)* eliminates the start codon of *wrn-1* and results in the complete absence of WRN-1 protein as determined by immunostaining germ cells with the specific WRN-1 antibodies that we generated (Figure S1B) and by Western blotting (data not shown). We next wished to determine the role of WRN-1 in relation to the previously established roles of ATL-1 (worm ATR), and CHK-1 in the DNA replication checkpoint by analyzing the morphology of DAPI-stained nuclei of germ cells after HU treatment (Figure S2A). In contrast to the wild type where germ cell nuclei were enlarged and had a diffuse distribution of chromatin in response to HU treatment, the majority of nuclei in *wrn-1(gk99)*, *atl-1(*RNA*i)*, or *chk-1(*RNA*i)* worms were small and apparently continued to divide. These nuclei had unusually compact chromatin and showed signs of extensive chromatin fragmentation, a phenotype likely to reflect escape from S-phase arrest and subsequent abnormal mitosis (Figure S2A). Inhibition by *chk-1* and *atl-1* RNA*i* was not complete but extensive, as determined by reverse transcription of mRNAs followed by quantitative PCR amplification (Figure S3A). In contrast, *atm-1(gk186)* cells, the nuclei were uniformly enlarged as in wild type cells, suggesting that ATM-1 is not required for the DNA replication checkpoint, in agreement with Garcia-Muse and Boulton [26]. The convergence of the *wrn-1(gk99)* and the *atl-1* and *chk-1* RNA*i* phenotypes suggested that *wrn-1*, *atl-1*, and *chk-1* might act in the same genetic pathway needed for HU-mediated cell cycle arrest. To test this hypothesis we asked if the phenotypes resulting from *atl-1* or *chk-1* RNA*i* were aggravated in the *wrn-1(gk99)* background and found that this was not the case (Figure S2A). Thus it is likely that *wrn-1* indeed functions in the same linear genetic pathway as *atl-1* and *chk-1* to mediate HU-dependent cell cycle arrest.

**Figure 2:**
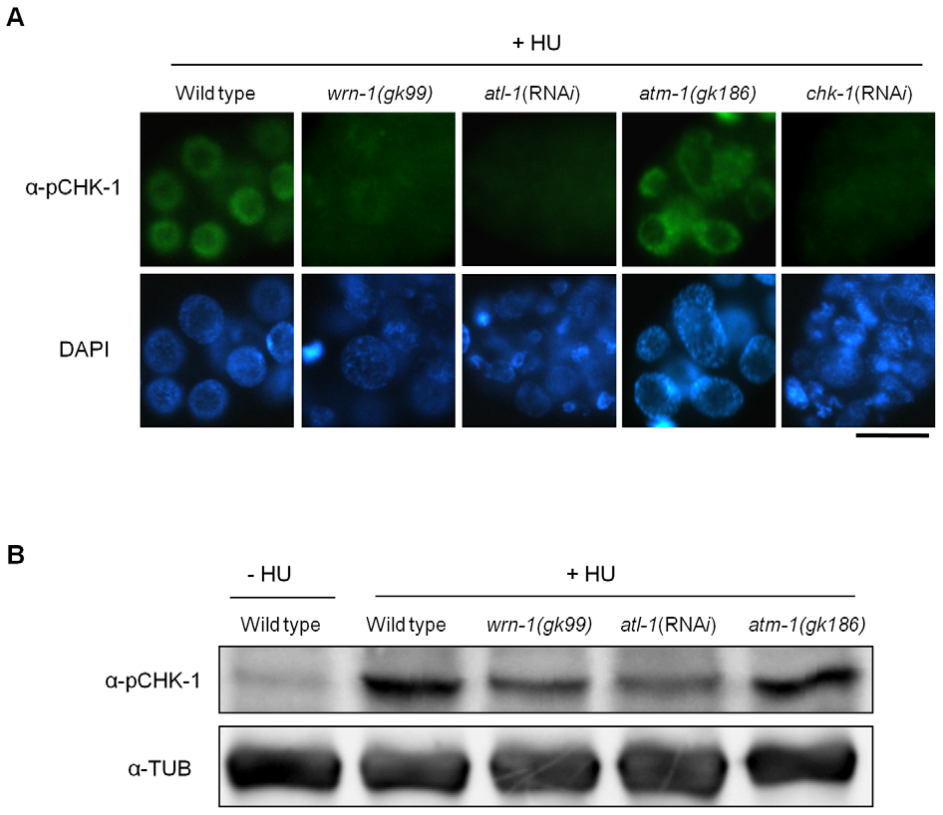
WRN-1 is required for the phosphorylation of CHK-1(S345) induced by inhibition of DNA replication. Wild-type N2, *wrn-1(gk99)*, *atl-1(*RNA*i)*, *atm-1(gk186)*, and *chk-1(*RNA*i)* worms were treated with 25 mM hydroxyurea (HU) from the L4 stage for 16 h. To knockdown *atl-1* (ATR homolog) or *chk-1* expression, worms were fed *E. coli* cells expressing the cognate double-stranded RNA from the L1 stage to the adult stage. (A) Phosphorylation of CHK-1(S345) in premeiotic germ cells probed with phospho-CHK1(S345) antibody. Magnification bar, 10 μm. (B) Worm extracts analyzed by western blotting using antibodies to phospho-CHK1(S345), and to α-tubulin as a control.

Having established that *wrn-1*, *atl-1*, and *chk-1* are required for activation of the intra S-phase checkpoint, we next tested if CHK-1 phosphorylation after HU treatment depends on WRN-1 (Figure 2A). We found that CHK-1 phosphorylation in germ cells was greatly reduced in a *wrn-1*(*gk99*) mutant (and also after RNA*i*-mediated *wrn-1* knockdown, data not shown) and after *atl-1* RNA*i* (Figure 2A). The dependence of CHK-1 phosphorylation on WRN-1 and ATL-1 after DNA replication inhibition was also confirmed by Western blotting of worm extracts with the pSer345 CHK1 antibody (Figure 2B), indicating that CHK-1 phosphorylation is reduced in *wrn-1* and *atl-1* in the extracts. This reduction is not as extensive as the reduction observed by the immunostaining of germ cells but nevertheless appears to reflect a reduction in CHK-1 phosphoprylation. CHK-1 protein levels are likely to be the same in wild-type, *wrn-1* or *atl-1* worms, given that we did not observe a difference in *chk-1* mRNA levels in those strains (Figure S3B). We could not directly confirm CHK-1 proteins levels due to the absence of a specific antibody (data not shown). Unlike the cases of *wrn-1* and *atl-1* deficiencies, the CHK1 phosphorylation was not affected by knockout of a *C. elegans* ATM homolog, ATM-1 (Figure 2A and 2B).

**Figure 3:**
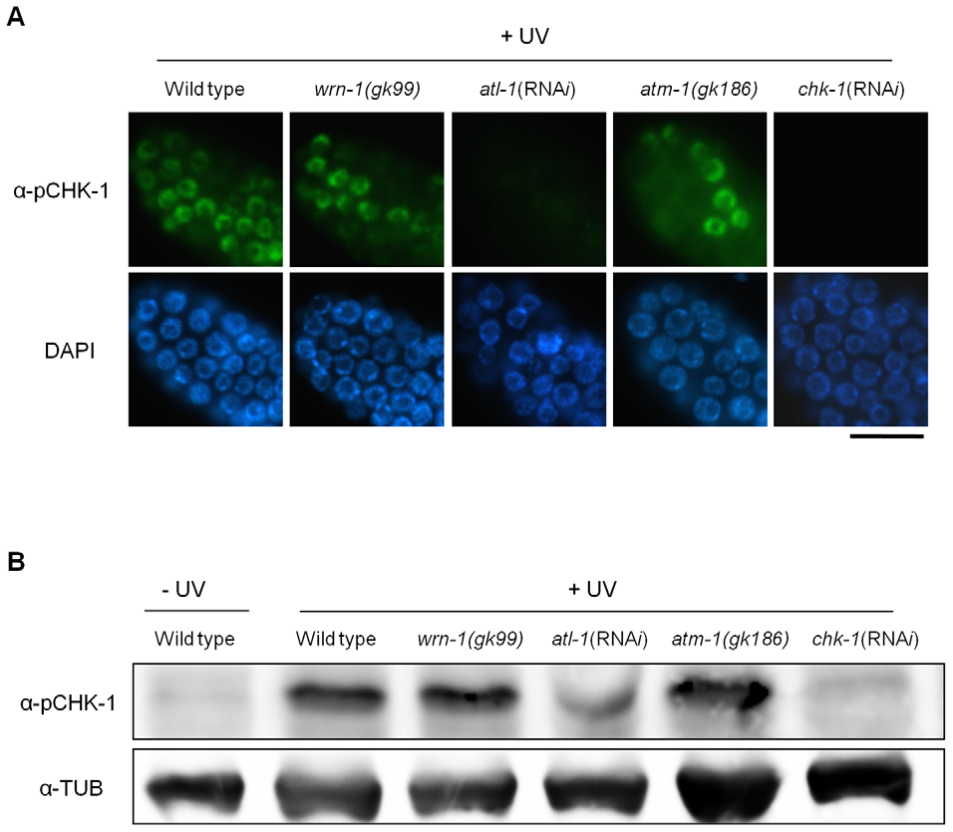
The *wrn-1* mutation does not affect the phosphorylation of CHK-1(S345) after UV-irradiation. Wild-type N2, *wrn-1(gk99)*, *atl-1(*RNA*i)*, *atm-1(gk186)*, and *chk-1*(RNA*i*) worms were irradiated as one-day-old adults with UV radiation (100 J/m^2^) and cultured for 1 h before immunostaining. *atl-1* and *chk-1* expression was knocked down as in Figure 2. Phosphorylation of CHK-1(S345) was probed as in Figure 2 by (A) immunostaining of mitotic germ cells and (B) western analysis of worm extracts. Magnification bar, 25 μm.

In contrast to the effect of DNA replication inhibition, CHK-1 phosphorylation caused by UV-radiation was not affected by the *wrn-1* mutation (Figure 3A), whereas it was greatly attenuated by *atl-1* RNA*i*. This observation in germ cells was confirmed by Western analysis of worm extracts (Figure 3B). In summary, our data suggest that WRN-1 functions upstream of CHK-1 in the DNA replication checkpoint but not in the DNA damage checkpoint activated by UV radiation. Either deficiency of *atm-1* or *wrn-1* was not detrimental enough to induce CHK-1 phosphorylation, in the absence of UV-radiation (Figure S4).

### WRN-1 Is Required for the Efficient Formation of RPA-1 Foci in Response to DNA Replication Inhibition

**Figure 4:**
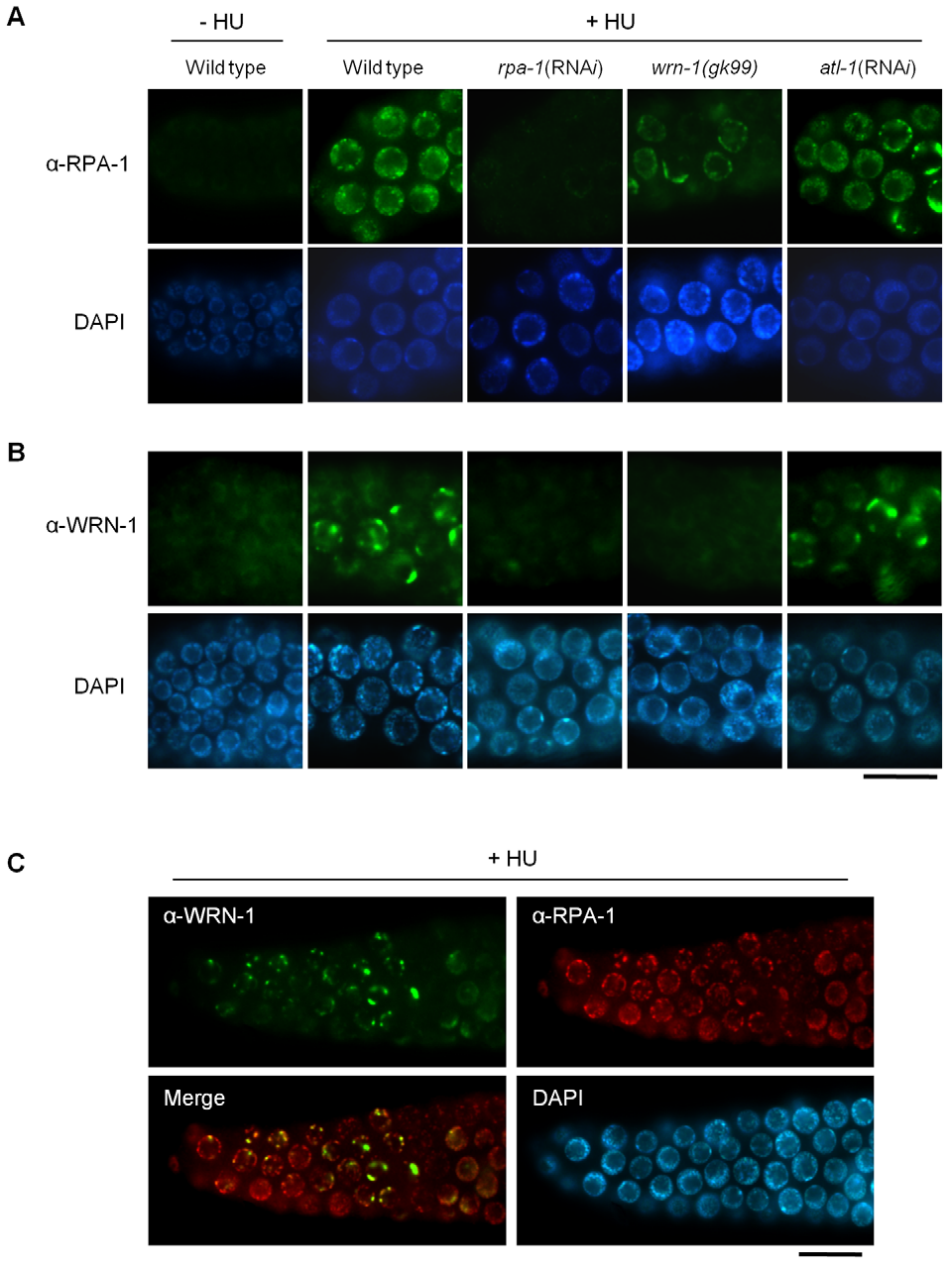
Reciprocal dependence of RPA-1 focus formation and nuclear localization of WRN-1 after DNA replication inhibition. One-day old adult wild-type N2, *rpa-1*(RNA*i*), *wrn-1(gk99)*, and *atl-1*(RNA*i*) worms were treated with hydroxyurea (25 mM) for 8 h. Knockdown of *atl-1* and *rpa-1* was carried out as in Figure 2 but from the L4 stage for 16 h, and premeiotic germ cells were stained with antibodies against (A) RPA-1 and (B) WRN-1. (C) Partial colocalization of RPA-1 and WRN-1 in the nuclei of premeiotic germ cells after hydroxyurea treatment. All the WRN-1 spots overlap with RPA-1 foci but not vice versa. Magnification bars, 10 μm.

Since WRN-1 controls CHK-1 phosphorylation after DNA replication inhibition, we asked whether RPA focus formation, which occurs upstream of CHK1 and ATR in mammalian cells, is also influenced by WRN-1. After HU treatment the nuclei of wild type cells were enlarged and contained RPA-1 foci, as demonstrated by Garcia-Muse and Boulton [26] (Figure 4A). RPA-1 foci disappeared, and RPA-1 protein (Figure S5C) in worm extracts was greatly reduced by *rpa-1* RNA*i* as well as the mRNA level (Figure S3A), (this also confirms efficient RPA-1 depletion and the specificity of our antibody). In *wrn-1(gk99)* germ cells, RPA-1 foci were not as abundant or intensely fluorescent as in wild type cells (Figure 4A and Figure S5A with and without HU treatment, respectively). However, RPA-1 focus formation was not affected by *atl-1* knockdown, as shown by Garcia-Muse and Boulton [26], and in agreement with the fact that in mammalian cells RPA binds to single-stranded DNA and then recruits ATR by binding to ATRIP [27]. In line with the results of Figure 2, *rpa-1* RNA*i* also largely eliminated the phosphorylation of CHK-1-Ser345 induced by HU (Figure S2B). The observation that the *wrn-1* mutation affects RPA-1 focus formation suggested that WRN-1 either functions upstream of RPA-1 or in the same step as RPA-1 focus formation. To distinguish between these possibilities, we tested whether nuclear localization of WRN-1 was affected by RPA-1. WRN-1 was present in the nuclei of all control germ cells and its abundance increased significantly after HU treatment (Figure 4B). In addition, the distribution of WRN-1 in the nucleoplasm became uneven, and WRN-1 aggregated into spots or lumps that were less abundant than RPA-1 foci in response to HU treatment. In *rpa-1* knockdown cells, neither the increase in WRN-1 abundance nor its aggregation into spots or lumps occurred upon HU treatment (Figure 4B and Figure S5B with and without HU treatment, respectively). At present, we can not completely rule out that the failure of CHK-1-Ser345 phosphorylation and WRN-1 spot formation upon RPA-1 depletion could be due to abolished DNA replication predicted to occur when RPA-1 is fully depleted. We consider that this possibility is unlikely, as we only partially depleted RPA-1 allowing a continued germ cell proliferation and DNA replication. Furthermore, we found that almost all WRN-1 nuclear spots colocalized with RPA-1 foci, but not vice versa (Figure 4C), further supporting the notion that RPA-1 may acts prior to or in the same step as WRN-1 at stalled replication forks.

### WRN-1 Participates Upstream of ATM-1 and RPA-1, but Downstream of MRE-11, in the Checkpoint Activation Induced by Ionizing Radiation

**Figure 5:**
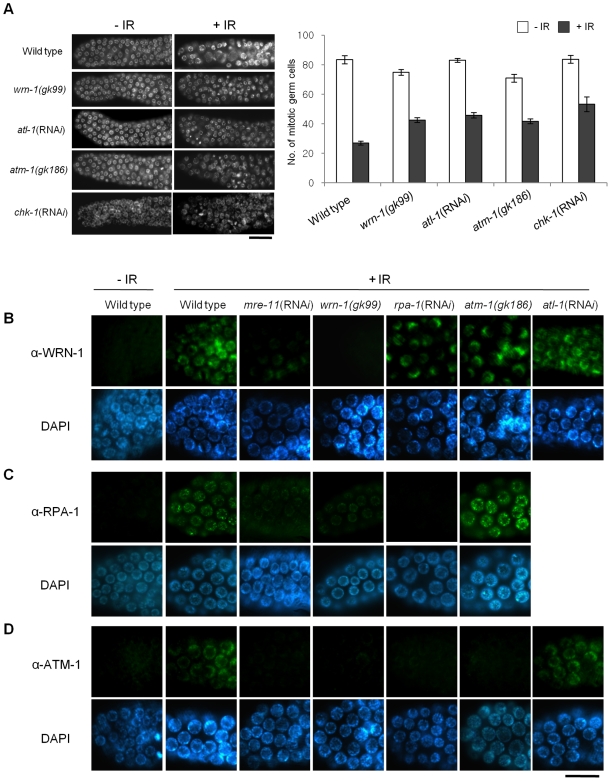
WRN-1 influences cell cycle arrest and the nuclear localization of ATM-1 and RPA-1 after γ-irradiation. (A) L4-stage wild-type N2, *wrn-1(gk99)*, *atl-1*(RNA*i*), *atm-1(gk186)* and *chk-1*(RNA*i*) worms were irradiated with γ-rays (75 Gy), and cultured for 12 h before scoring germ cells. Germ cells in the mitotically proliferating region of gonads (within 75 µm of the tip cell) were stained with DAPI and counted under a fluorescence microscope. Error bars indicate standard errors of means of 12 worms of each genotype. (B–D) One-day-old adult worms of wild-type N2, *wrn-1(gk99)*, *atl-1*(RNA*i*), *atm-1(gk186)*, *chk-1*(RNA*i*), *rpa-1*(RNA*i*), and *mre-11*(RNA*i*) strains were irradiated with γ -rays (75 Gy) and cultured for 1 h before immunostaining. Mitotic germ cells were stained using antibody against (B) WRN-1, (C) RPA-1, and (D) ATM-1. Knockdown of *atl-1, chk-1, mre-11*, and *rpa-1* were carried out from (A) the L1 and (B–D) L4 stages. Magnification bars, (A) 25 µm and (B–D) 10 µm.

We next wished to test if *wrn-1* functions in the checkpoint pathway that leads to cell cycle arrest of γ-irradiated proliferating germ cells. After γ-irradiation, the number of germ cells in the mitotic region of gonads is greatly reduced in wild type worms (Figure 5A) due to cell cycle arrest. Arrested cells, however, enlarge due to continued cellular growth in the absence of cell division [18]. We found that in response to IR the number of mitotic germ cells was much less reduced in *wrn-1(gk99)*, *atl-1*(RNA*i*), *atm-1(gk186)*, and *chk-1*(RNA*i*) gonads as compared to wild type gonads, suggesting that WRN-1 and the three other well-known checkpoint proteins play significant roles in IR-dependent cell cycle arrest (Figure 5A). To determine the epistatic relationships of WRN-1 in this checkpoint signaling pathway, we examined whether the nuclear localization of WRN-1 was altered in response to IR and if so whether this depended on various known DNA damage checkpoint and repair proteins. We found that WRN-1 accumulated on the chromatin of irradiated wild type cells and that this accumulation was greatly attenuated by *mre-11*(RNA*i*), but was unaffected by *rpa-1*(RNA*i*), *atl-1*(RNA*i*), and the *atm-1(gk186)* mutation (Figure 5B and Figure S6A with and without IR, respectively) (the RNA*i* efficiencies were confirmed in Figure S3A). Since *C. elegans* RPA-1 was reported to form nuclear foci in response IR [26], we tested whether their formation was affected by WRN-1. In fact, IR-induced RPA-1 focus formation was significantly reduced by the *wrn-1(gk99)* mutation (Figure 5C). In contrast, RPA-1 focus formation was more evident in the *atm-1* mutant than in wild type cells, indicating that *atm-1* is not required for RPA-1 focus formation, a result in agreement with the previous report by Garcia-Muse and Boulton [26]. We next asked if ATM accumulates on chromatin in response to IR and if so whether this depends on *wrn-1*. For this purpose, we generated a specific ATM-1 antibody and found that ATM accumulated on the chromatin of wild type cells in response to IR treatment (Figure 5D). However, it did not accumulate significantly upon IR treatment of *mre-11*(RNA*i*), *wrn-1(gk99)*, or *rpa-1*(RNA*i*) cells, implying that the IR-dependent accumulation of ATM on chromatin requires the products of all three genes (Figure 5D and Figure S6B with and without IR, respectively). In contrast, *atl-1* knockdown did not affect ATM-1 localization (Figure 5D), suggesting that ATM-1 acts in a parallel or independent pathway. Our combined results therefore suggest that sequential action of MRE-11 and WRN-1 is needed for efficient RPA-1 focus formation, and that this in turn leads to the nuclear accumulation of ATM-1 in response to IR.

## Discussion

**Figure 6:**
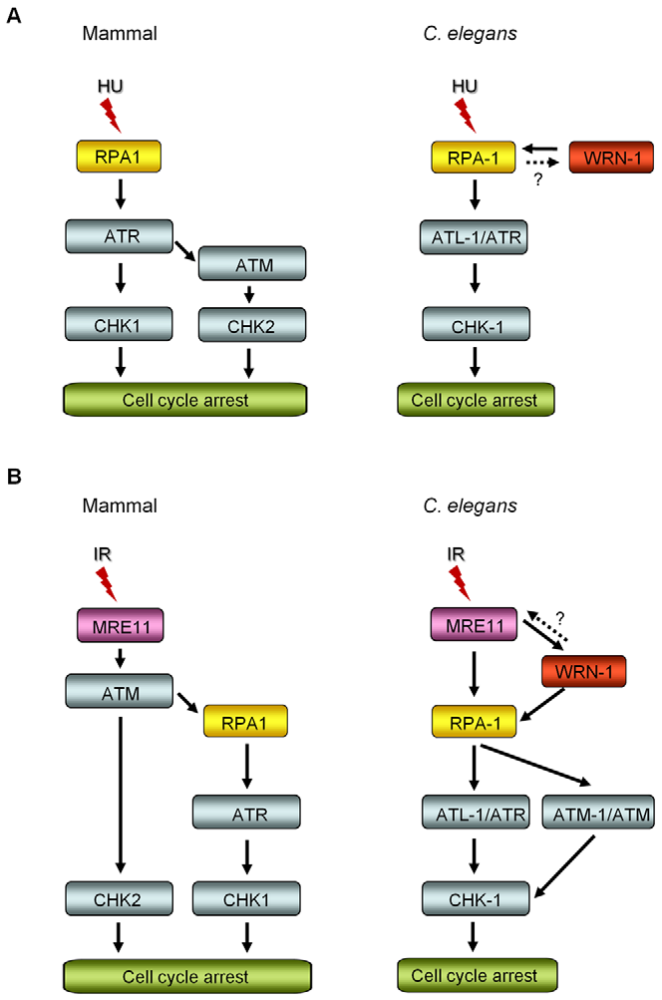
Comparison of DNA replication and damage checkpoint pathways between mammals and *C. elegans*, and roles of the *C. elegans* WRN homolog in these pathways. (A) After DNA replication inhibition by hydroxyurea (HU), ATM-CHK2 activation is induced in the downstream of ATR in mammals, in addition to ATR-CHK1 activation [51]. In *C. elegans*, the nuclear focus formation of RPA-1 and WRN-1 are interdependent, however RPA-1 knockdown may have indirectly affected WRN-1 foci by reducing the number of replication forks. ATR/ATL-1 is inserted between RPA-1 and CHK-1, because it was positioned below RPA-1 by Garcia-Muse and Boulton [26]. (B) Ionizing radiation (IR) induces ATR-CHK1 activation in the downstream of ATM, as proposed by Jazayeri et al. [44] in mammals. In *C. elegans*, the nuclear localization of WRN-1 in response to IR is affected by MRE-11 and is a prerequisite for efficient RPA-1 focus formation. It needs to be determined whether WRN-1 conversely affects the nuclear localization or function of MRE-11, as labeled by a question mark. The nuclear accumulation of ATM requires WRN-1 and RPA-1, as well as MRE-11. ATR/ATL-1 is positioned below RPA-1, as proposed by Garcia-Muse and Boulton [26], and is required for effective cell cycle arrest (Figure 5A). CHK-1 is located below ATR, because it was shown to be essential for cell cycle arrest in response to IR by Kalogeropoulos et al. [52] and also in Figure 5A.

We have demonstrated that WRN-1 regulates the DNA replication checkpoint upstream of CHK-1, and probably in the same step as RPA-1, but that the checkpoint activation induced by UV is not affected by WRN-1. It is an intriguing question whether the helicase activity of WRN-1 is required for the DNA replication checkpoint. We have observed normal cell cycle regulation after HU- or IR-treatment of *wrn-1(tm764)* mutants, which have a deletion from the fifth exon to the following intron (Figure S1A, S1B, S1C, S1D). Since this deletion eliminates the helicase motif, it is likely to abolish the helicase activity of wild type WRN-1 that was measured by Hyun et al. [28] in vitro. Therefore, the helicase activity of WRN-1 seems not to be essential for checkpoint function. However, this activity is probably essential for the function of WRN-1 in DNA repair, since the *wrn-1(tm764)* as well as the *wrn-1(gk99)* mutation resulted in increased frequency of developmental defects in response to IR (Figure S1E, S1F). A similar observation was made for SGS1, the only RecQ helicase in *S. cerevisiae*: its helicase activity was not required for activation of RAD53, a CHK2 homolog [29], after inhibition of DNA replication. Among five RecQ homologs in humans, only limited checkpoint activity has been observed for WRN, namely upon inhibition of chromosomal decatenation [15] and in the activation of ATM induced by interstrand DNA crosslinking and DNA replication inhibition [16]. We propose that WRN-1 affects CHK-1, and probably also ATL-1/ATR activation, by increasing the stability of RPA on the single stranded DNA (ssDNA) at arrested DNA replication forks (Figure 6A). While the components of the so called 9-1-1 complex, implicated in recognizing arrested replication forks are conserved in *C. elegans* [30]–[32], the gene coding for ATRIP, which recruits ATR to RPA on ssDNA, has not been identified in *C. elegans*. WRN-1 may be able to substitute for ATRIP and recruit ATL-1/ATR to the fork, a hypothesis supported by the finding that RPA physically interacts with WRN [28],[33] and by the colocalization of WRN-1 and RPA-1 (Figure 4C). However, at present we do not know if *C. elegans* WRN-1 physically interacts with RPA and ATL-1/ATR in vivo. The positioning of WRN-1 in the same step or upstream of RPA-1 in our model differs from the situation in human cells, where depletion of WRN does not affect fork recovery but impairs fork progression after replication inhibition [34]. Another possible function of WRN-1 is to promote the uncoupling of DNA polymerase and helicase activities at stalled replication forks, thereby increasing the length of ssDNA and the concomitant coating of the ssDNA with RPA-1 [35]. Although we place WRN-1 in the same step as RPA-1, upstream of ATR and CHK1, WRN-1 is not as essential for worm survival under normal conditions as these three checkpoint proteins. A possible reason for this difference is that WRN-1 may only respond to exogenously induced stalls of replication forks, but not to those formed spontaneously. In agreement with this idea, RAD-51 foci, indicative of replication fork collapse, were not observed in *wrn-1(gk99)* cells in the absence of exogenously-induced DNA damage (data not shown), whereas they were observed in the *atl-1(tm853)* mutant [26]. Unlike the case of DNA replication inhibition, WRN-1 is not needed for the checkpoint activation induced by UV; this could be due to the ability of ATR to bind to UV-damaged DNA [36] or due to the presence of another protein that recruits ATR to UV-damaged DNA.

We found that in contrast to wild type, the cell cycle arrest was significantly alleviated in *wrn-1* mutants by treatment with 75 Gy of IR, as well as in *atl-1*, *atm-1*, and *chk-1* deficient animals (Figure 5A). Stergiou et al. [37] found that the *atm-1* mutation did not affect the cell cycle arrest induced by high dose IR (120 Gy), but they noted a moderate effect on the apoptosis of germ cells after low dose IR (20–40 Gy). However, another study based on *atm-1* RNA*i* reported that ATM-1 is needed for IR-dependent cell cycle arrest upon treatment with 75 Gy of IR [26].

Our results suggest that WRN-1 is an upstream component of the signaling pathway that mediates IR-dependent cell cycle arrest. We found that in response to IR the nuclear localization of WRN-1 depended on the presence of MRE-11 to a significant extent (Figure 5B), and that RPA-1 focus formation was promoted by WRN-1 (Figure 5C). In human cells, WRN interacts with the MRN complex via the NBS1 subunit of MRN, and WRN focus formation depends on NBS1 [13],[14]. Nevertheless, it is likely that the accumulation of *C. elegans* WRN-1 is regulated differently, as there is no NBS1 homolog in the worm genome. However, the fact that WRN translocates to double-strand DNA breaks (DSBs) within a few minutes, with the same kinetics as NBS1, also agrees with the proposed role of WRN-1 at the initial stage of the checkpoint pathway [38].

We propose that WRN-1 functions downstream of MRE-11 and upstream of RPA-1 in the DSB signaling pathway leading to cell cycle arrest, as illustrated in Figure 6B. Nevertheless, it is not yet clear whether WRN-1 also affects the nuclear localization or function of MRE-11. Recently, the MRN complex, CtIP, and Exo1 nucleases were found to be involved in the resection of DSBs to produce ssDNA in vertebrate cells [39]–[41], the process that initiates ATR-mediated DNA damage signaling [42] and homologous recombination: a RecQ family helicase BLM participates in the DSB resection as a partner of Exo1 [43]. Likewise, *C. elegans* WRN-1 may affect the activity of MRE-11 nuclease, which could explain the stimulation of RPA-1 focus formation by WRN-1. In the model of DNA damage signaling pathway depicted in Figure 6B, ATM-1 is located downstream of RPA-1, unlike ATM in mammals which functions upstream of RPA [44]. However, another single-stranded DNA binding protein, hSSB1, was recently found to be essential for ATM activation in response to IR [45]. We therefore speculate that the role of RPA-1 in activating ATM in *C. elegans* may have been acquired by hSSB1 in vertebrates.

Ionizing radiation (IR) did not induce CHK-1-Ser345 phosphorylation in mitotically proliferating germ cells (Figure 1A). However, CHK-1 was required for the efficient inhibition of germ cell proliferation upon IR (Figure 5A) in accord with previous reports. The absence of CHK-1-Ser345 phosphorylation (Figure 1B) is in line with a previous report showing that Ser345 of CHK1 is not phosphorylated by IR in mammalian cells whereas Ser317, which is not conserved in *C. elegans*, is phosphorylated [23]. We thus hypothesize that some other residue in *C. elegans* CHK-1 is phosphorylated after IR to activate the protein.

In summary, the *C. elegans* homolog of human WRN helicase, WRN-1, regulates the DNA damage signaling pathway induced by DNA replication inhibition and double-stranded DNA breaks upstream of ATR, probably at or close to the step involving RPA localization, and upstream of ATM. Human WRN was recently found to be essential for the activation of ATM after treating cells with UV-activated psoralen and hydroxyurea, but ATM activation induced by γ-rays was not dependent on WRN [16]. These results in humans differ from our findings in *C. elegans*, where WRN-1 is required for the nuclear accumulation of ATM induced by DSBs, as well as for activation of ATR and CHK1 at stalled replications forks. Presumably, the relative levels of key proteins involved in DNA damage signaling vary between organisms, tissues, and cell types, so that the extent of the contribution of various proteins of each (sub)pathway is likely to differ between organisms. We therefore consider that the upstream DNA damage signaling functions of WRN-1 uncovered here are likely to be conserved, but might be cryptic in human systems due to functional redundancy. It will be interesting to follow up this possibility in future studies and to ask how *C. elegans* WRN-1 recruits and activates RPA and ATM in response to checkpoint activation.

## Materials and Methods

### Strains and EST Clones

*C. elegans* strain Bristol N2 was maintained as described [46] at 20°C unless noted. The *wrn-1(gk99)* and *atm-1(gk186)* strains were obtained from the *C. elegans* Genetics Center (St Paul, MN, USA). *wrn-1(tm764)*, which was generated as part of the National Bioresource Project (Japan), was obtained from Dr. Shohei Mitani (Tokyo Women's Medical University School of Medicine). The mutant strains were outcrossed six times with N2 to remove possible unrelated mutations. The EST clones of *atl-1* (yk1218d05), *chk-1* (yk1302e07), *rpa-1* (yk787c12), and *mre-11* (yk133b9) were provided by Dr. Y. Kohara (National Institute of Genetics, Japan).

### Bacteria-Mediated RNA*i*

Bacteria-mediated RNA*i* of *chk-1*, *atl-1*, *rpa-1*, and *mre-11* was performed as described, with minor modifications [47]. An approximately 1.2 kb cDNA fragment of the open reading frame (ORF) of Y39H10A.7 (CHK-1) was derived from the EST clone yk1302e07 after being digested with *Xho*I. The EST clone yk1218d05 of *atl-1* was digested using *BamH*I and *Pst*I to produce a 1.1 kb cDNA fragment. The EST clone yk787c12 of *rpa-1* was digested using *Xho*I to produce a 0.64 kb cDNA fragment, and yk133b9 of *mre-11* using *Xho*I and *Xba*I to produce a 1.6 kb fragment.

The cDNA fragments were cloned into pPD129.36(L4440) plasmid and transformed into *Escherichia coli* strain HT115(DE3). Ten N2 worms were allowed to lay embryos on plates covered with *E. coli* cells producing double-stranded RNA of targeted genes for 2 h. F1 worms were grown on RNA*i* feeding plates to the L4 stage or 1-day-old adults at 25°C before being treated with DNA damaging agents. In the experiments shown in Figure 4 and Figure 5B–5D), RNA*i* was performed for 16 h from the L4 stage (instead of the L1 stage) before treatment with HU or IR.

### Hydroxyurea Treatment

To observe pCHK1 expression or nuclear morphology, worms were grown from L1 to L4 larvae on RNA*i* feeding plates at 25°C. L4 stage worms were transferred to new RNA*i* feeding plates containing 25 mM hydroxyurea. After 16 h, their gonads were dissected out and immunostained using rabbit-anti-phospho-CHK1(Ser345) antibody. To visualize WRN-1 and RPA-1 expression, worms were grown from L4 larvae to 1-day-old adults (for 16 h) on RNA*i* feeding plates at 25°C, and then transferred to RNA*i* feeding plates containing 25 mM hydroxyurea for 8 h. Dissected gonads were immunostained with anti-WRN-1 and anti-RPA1 antisera.

### γ- and UV Radiation

Worms were grown from L1 larvae to 1-day-old adults at 25°C on RNA*i* feeding plates (except for Figure 5B–5D, where RNA*i* was performed from the L4 stage for 16 h). They were γ-irradiated (75 Gy) using a ^137^Cs source (IBL 437C, CIS Biointernational) or UV-irradiated (100 J/m^2^) using a CL-1000 UV crosslinker (Ultra-Violet Products). After 1 h, gonads were dissected and immunostained.

### Antibody Preparation

Polyclonal antiserum to WRN-1 protein was generated by immunizing mice and rabbits with an amino-terminal WRN-1 fragment corresponding to amino acids 1–209, as described in Lee et al. [19]. The mouse and rabbit anti-WRN-1 polyclonal antibodies were used for immunostaining and Western blot analysis, respectively.

The 1–699 bases of the *rpa-1* open reading frame (ORF) were amplified by PCR from EST clone yk787c12 with forward primer 5′-CACCATGGCGGCAATTCACATCAATCAC and reverse primer 5′-TACGTACGGTGTAACCATTGA. An *atm-1* cDNA fragment containing 748–1950 nucleotides of the ORF was prepared from EST clone yk444h6 by PCR with forward primer 5′-CACCGAAATTGCAATGCTTGACG and reverse primer 5′-CTACAAAAACGGCATCCAT. The amplicons were subcloned into pENTR/D/TOPO (Invitrogen). The constructs were subsequently cloned into pDEST15 using the Gateway cloning system (Invitrogen). pDEST15 recombinants containing the *rpa-1* or *atm-1* fragment were transformed into *E. coli* BL21AI. The *E. coli* cells were grown at 37°C to an OD_600 nm_ of 0.5 in LB medium containing 100 *µ*g/mL ampicillin. L-arabinose (Sigma-Aldrich) was added to a final concentration of 0.2% (w/v), and the cells were grown for an additional 4 h at 37°C. The overexpressed proteins were used to generate antibodies in rats. Rabbit anti-CHK1(pSer345), mouse anti-α-tubulin, and anti-BrdU antibodies were purchased from Cell Signaling Technology, Developmental Studies Hybridoma Bank, and Becton-Dickinson, respectively.

### Immunostaining

Gonads were extruded by decapitating adult worms, fixed in 3% paraformaldehyde, and immunostained as described [48]. However, to enhance immunostaining of phospho-CHK1 and WRN-1, we used a tyramide signal amplification system (Invitrogen). Gonads were reacted with rabbit anti-CHK1(pSer345) or mouse anti-WRN-1 (1∶50 dilution), followed by horseradish peroxidase (HRP)-goat anti-rabbit (or anti-mouse) IgG at 1∶100 dilution. Alexa Fluor 488 tyramide at 1∶100 dilution was used to detect HRP. Anti-RPA-1 and anti-ATM-1 antisera (1∶50 dilution) were incubated with gonads, which were then reacted with Fluor 488-conjugated goat anti-rat secondary antibodies (Molecular probes, 1∶1000 dilution). When mouse anti-BrdU antiserum (1∶10 dilution) was used as a primary antibody, Alexa Fluor 555-conjugated goat anti-mouse antibody (Molecular Probes) was used as the secondary antibody. After staining with DAPI (4,6-diamidino-2-phenylindole, 1 mg/ml), specimens were observed with a fluorescence microscope (DMR HC, Leica).

### BrdU Labeling and Immunostaining

To incorporate BrdU into *E. coli* chromosomal DNA, *E. coli* MG1693 cells, which are thymidine auxotrophs (from *E. coli* Genetic Stock Center), were grown overnight in LB containing 10 µg/ml trimethoprim, 0.5 µM thymidine, and 10 µM BrdU, as described by Ito and McGhee [49]. *C. elegans* chromosomal DNA was labeled with BrdU by culturing hermaphrodites for 30 min on NGM plates seeded with BrdU-labeled *E. coli* cells. Adult worms were treated with γ- or UV radiation and transferred to plates containing unlabeled *E. coli* OP50 cells. After 1 h, gonads were extruded, fixed and washed, and then soaked in 2 N HCl for 15 min at room temperature to denature DNA and expose the BrdU epitope. This was followed by neutralization in 0.1 M sodium tetraborate solution for 15 min at room temperature and double immunostaining for pCHK-1 and BrdU.

### Western Blot Analysis

Adult worms were washed off ten NGM plates (dia. 55 mm) with 1×PTW (PBS with 0.1% Tween 20). The pellets were mixed with 2 volumes of 2× sample loading buffer (200 mM Tris⋅Cl, pH 8.0, 500 mM NaCl, 0.1 mM EDTA, 0.1% Triton X-100, 0.4 mM PMSF) and kept in boiling water for 10 min. The lysates were electrophoresed on 8% SDS polyacrylamide gels and electroblotted onto nitrocellulose membranes (Schleicher & Schuell BioScience). The antibody dilutions were 1∶1000 for phospho-CHK1, WRN-1, and RPA-1, and 1∶10,000 for α-tubulin. Secondary anti-rabbit, anti-mouse or anti-rat HRP (Jackson Bioresearch, 1∶1000 dilution) antibody was used and detected using ECL (Amersham Sciences). Luminescence was captured with a LAS-3000 imaging system (Fujifilm).

### Assessment of Cell Cycle Arrest

L4 larvae were treated with 75 Gy of γ-rays, and their gonads were dissected out 12 h later. After staining with DAPI, the gonads were observed using a fluorescence microscope. The cell cycle arrest phenotype was assessed by counting the number of mitotic nuclei present in one focal plane within 75 µm of the distal tip cell.

### Measurement of mRNA by quantitative RT-PCR

To determine RNA*i* efficiency targeting *chk-1*, *mre-11*, *rpa-1*, and *atl-1*, worms were grown from L4 larvae to 1-day-old adults (for 16 h) on RNA*i* feeding plates at 25°C (Figure S3A). To test whether *atl-1* RNA*i* indirectly affected the level of *chk-1* mRNA, worms were grown from L1 to L4 larvae on the feeding plates at 25°C (Figure S3B). L4 stage worms were transferred to new RNA*i* feeding plates containing 25 mM hydroxyurea and grown for 16 h (Figure S3B). The worms were washed three times in 1× PTW (PBS with 0.1% Tween 20) to free them of bacteria. RNA extraction and quantitation of relative mRNA levels were performed as described previously [50]. Primers used for amplification (in 5′ to 3′ orientation) were;

*chk-1*: GGAGAGACAGAATGCTTCG, GACATCCACATTGATCGAG

*mre-11*: GAGTATGGGACAAGTTTCTGCA, ATCCCTTTTCTTGGATGGAGC

*atl-1*: TATCCAGAAGCAGGGCAATGG, TTAGAGGGTCGCCATCCAACC

*rpa-1*: ATGGCGGCAATTCACATCAATCAC, TACGTACGGTGTAACCATTGC

*tbb-1* (β-tubulin as a control): TCCTTCTCGGTTGTTCCATC, GGGAATGGAACCATGTTGAC.

## Supporting Information

Figure S1 Structures of *wrn* alleles, and comparison of their responses to hydroxyurea and ionizing radiation. (A) Schematic representation of the structure of the *C. elegans wrn-1* gene and of the deletions in the *gk99* and *tm764* alleles. (B) Representative images of the mitotic regions of wild-type, *wrn-1(gk99)*, and *wrn-1(tm764)* gonads from one-day-old adult worms immunostained with WRN-1 antibody before and after hydroxyurea (HU, 25 mM) treatment for 8 h. In the nuclei of *wrn-1(tm764)* gonads, WRN-1 is significantly increased by HU treatment, but not to the same level as in wild-type gonads. (C) Morphological changes of DAPI-stained nuclei in mitotic germ cells after HU treatment. After HU treatment, the premeiotic nuclei in wild-type and *wrm-1(tm764)* gonads were substantially enlarged and reduced in number. In contrast, much smaller nuclei, some of which were condensed, were observed in the *wrn-1(gk99)* and *wrn-1*(RNA*i*) gonads. Magnification bars are (B) 10 µm and (C) 25 µm. (D) L1-stage worms were irradiated with γ-rays (20 Gy) and their growth was measured after 48 h at 20°C. (E) Developmental abnormalities were scored 3 days after IR (60 Gy) at the L1 stage. Small, small body; Ruptured, ruptured body; Transp, transparent; Unc, uncoordinated movement. (F) Hatching rates were measured for F1 embryos collected 0–24, 24–48, and 48–72 h periods after exposing L4 stage worms of the P0 generation to IR (75 Gy).
(0.33 MB PDF)

Figure S2 Epistatic relationships of WRN-1 with checkpoint proteins in the cell cycle arrest induced by hydroxyurea. (A) Knockdown of *wrn-1*, *atl-1*, and *chk-1* were performed from the L1 stage. Images are DAPI-stained nuclei in the gonads of worms, untreated (−HU) or exposed to 25 mM hydroxyurea (+HU) from the L4 stage for 16 h. After HU treatment, premeiotic nuclei in wild-type and *atm-1(gk186)* gonads were substantially enlarged and reduced in number. In contrast, much smaller nuclei, some of which were condensed, were observed in the mitotic regions of *wrn-1*(RNA*i*), *atl-1*(RNA*i*), and *chk-1*(RNA*i*) gonads. Double deficiencies of *atl-1* or *chk-1* in the *wrn-1(gk99)* background did not increase the nuclear phenotype, compared with the single deficiencies of *atl-1*, *chk-1*, and *wrn-1*. *wrn-1*(RNA*i*) in the background of *wrn-1(gk99)* did not change the nuclear phenotype, supporting that *wrn-1(gk99)* is a null mutation. (B) Knockdown of *rpa-1* was carried out from the L4 stage for 16 h before HU treatment, and premeiotic germ cells were probed with phospho-CHK1(S345) antibody. *rpa-1* knockdown induced the nuclear phenotype as for the knockdown of *atl-1* or *chk-1*, and abolished phosphorylation of CHK-1(S345). Magnification bars are 25 µm.
(1.23 MB TIF)

Figure S3 Efficient knockdown of target mRNA expressions and no significant effects of other deficiencies on the *chk-1* mRNA expression. Total RNA was isolated form worms after performing feeding RNA*i* for 16 h. Reverse transcription after random priming and real-time PCR using gene-specific primers were followed. (A) The mRNA levels of target genes (*chk-1*, *mre-11*, *rpa-1*, and *atl-1*) were estimated by analyzing cDNA amplication kinetics, normalized to that of β-tubulin, and plotted as percent ratios of the corresponding value of the wild-type. The error bars indicate standard errors of the mean. The amplified cDNA fragments were separated on 0.7% agarose gels after 25 PCR cycles. (B) No significant effects of *wrn-1* or *atl-1* deficiency on the mRNA expression of *chk-1* (p values of t test>0.5). The *chk-1* mRNA levels were estimated as in (A) before and after HU treatment.
(0.32 MB TIF)

Figure S4 Absence of CHK-1(S345) phosphorylation in untreated germ cells deficient in WRN-1 or checkpoint proteins. (A) Phosphorylation of CHK-1(S345) in mitotic germ cells probed with phospho-CHK1(S345) antibody. Magnification bar, 10 µm. (B) Worm extracts analyzed by western blotting using antibodies to phospho-CHK1(S345), and α-tubulin as a control.
(0.97 MB TIF)

Figure S5 Absence of RPA-1 and WRN-1 focus formation in untreated germ cells, and specificity of RPA-1 antibody. Absence of (A) RPA-1 focus formation and (B) WRN-1 spot formation in germ lines deficient in *rpa-1*, *wrn-1*, or *atl-1* before hydroxyurea (HU) treatment. (C) Western analysis of RPA-1 in worm extracts after the knockdown, and of α-tubulin as a control. Magnification bar, 10 µm.
(1.34 MB TIF)

Figure S6 No significant effects of checkpoint proteins on the nuclear localization of WRN-1 and ATM-1 in untreated gonads. (A) Lack of significant effects of *mre-11* or *atm-1* deficiency on the nuclear localization of WRN-1, and (B) of *mre-11*, *wrn-1*, or *rpa-1* deficiency on the nuclear localization of ATM-1 in untreated gonads. However, *atl-1* deficiency slightly induced the nuclear localization of ATM-1 in untreated gonads, but the level reached was much lower than after IR. Worms were irradiated as one-day-old adults with IR (75 Gy) and cultured for 1 h before immunostaining. Magnification bars, 10 µm.
(1.43 MB TIF)
