# Supplementary figures and images for: The Caenorhabditis elegans Werner Syndrome Protein Functions Upstream of ATR and ATM in Response to DNA Replication Inhibition and Double-Strand DNA Breaks
Source: PLoS Genet. 2010 Jan 8;6(1):e1000801. doi: 10.1371/journal.pgen.1000801 (PMC2791846; doi:10.1371/journal.pgen.1000801)

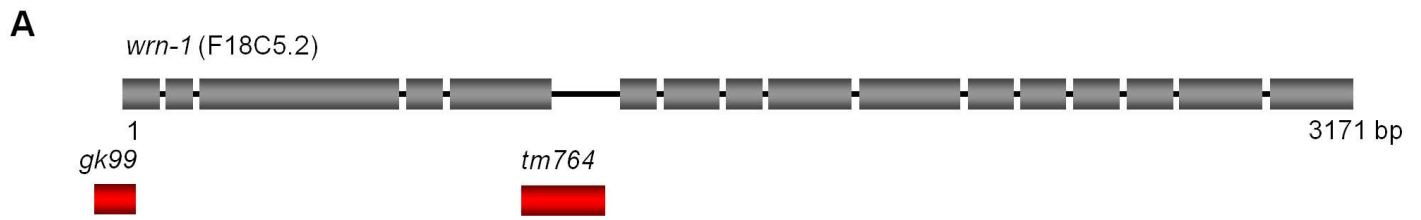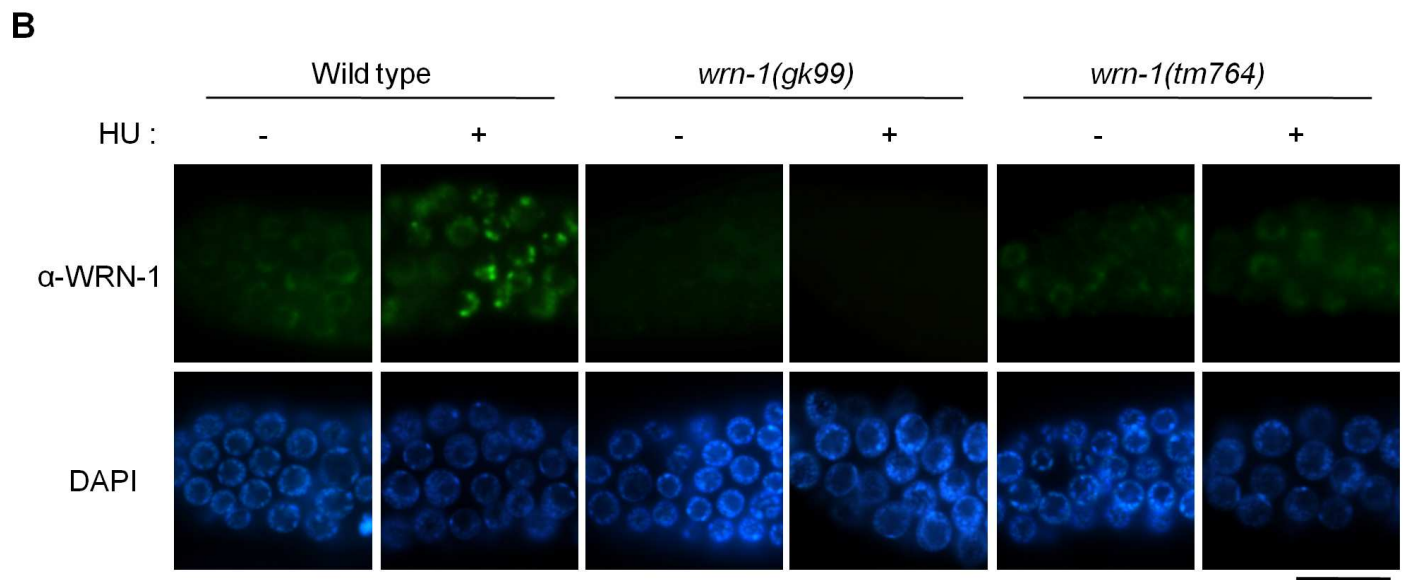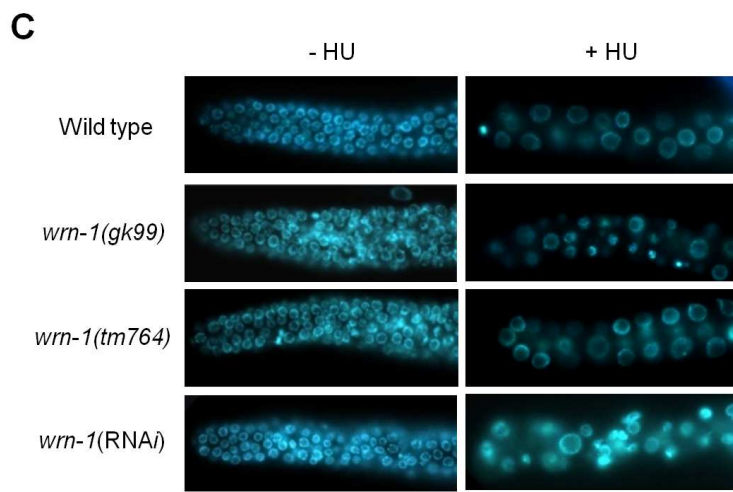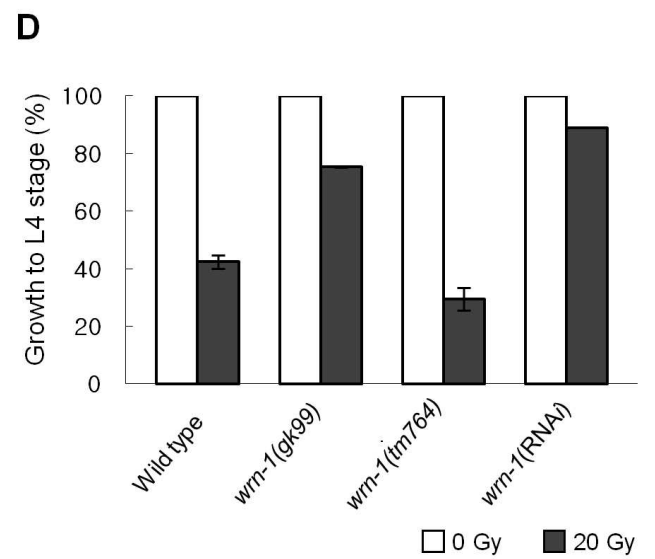

**E**

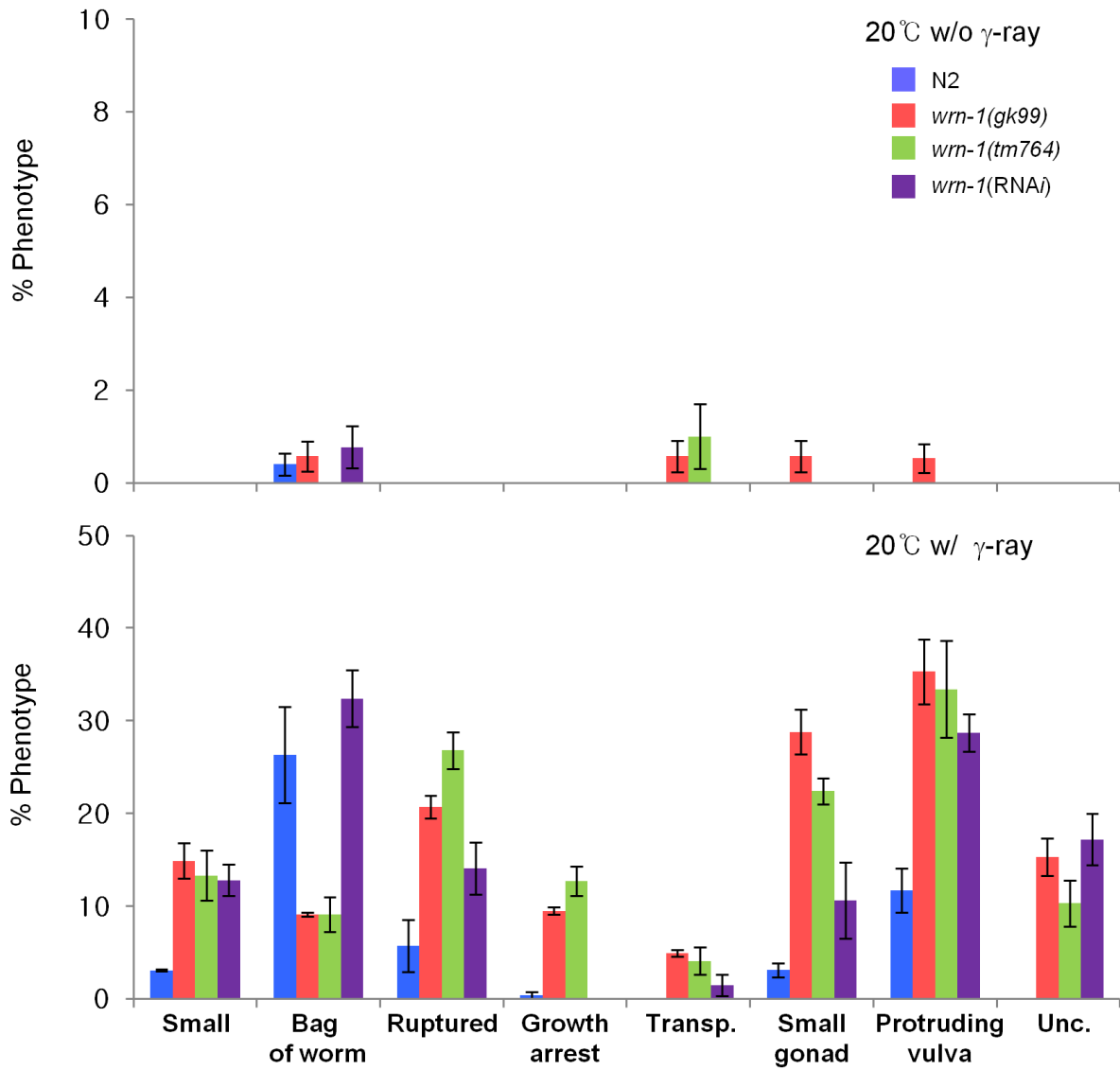

**F**

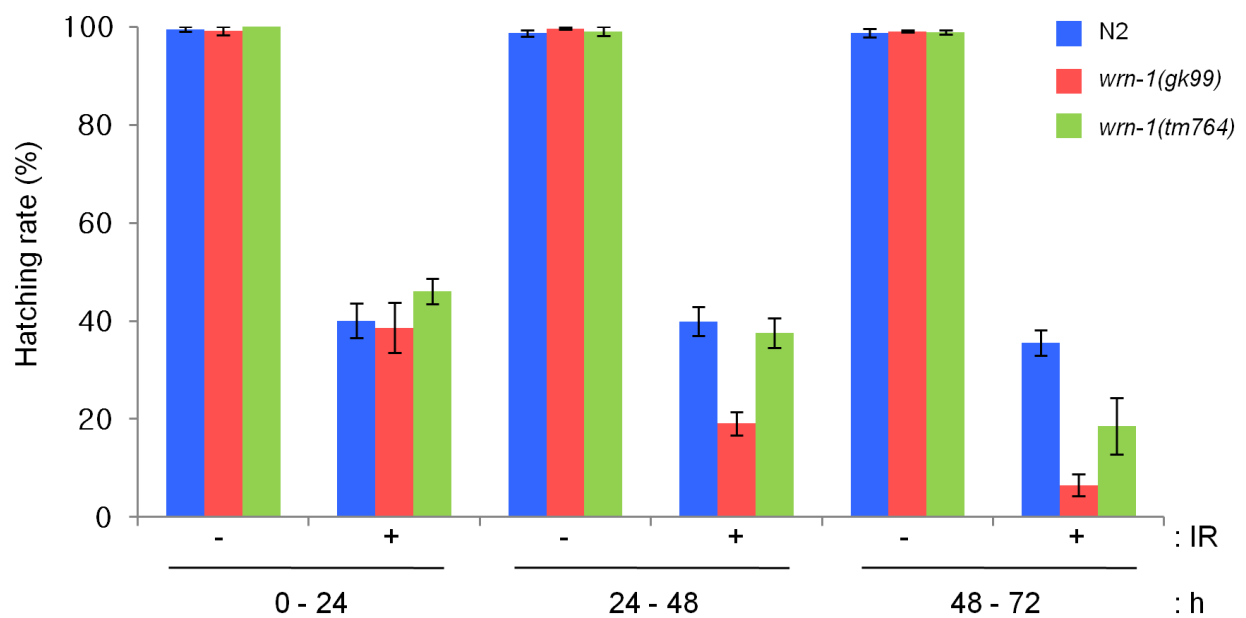

Supplement: Figure S1 — Structures of wrn alleles, and comparison of their responses to hydroxyurea and ionizing radiation. (A) Schematic representation of the structure of the C. elegans wrn-1 gene and of the deletions in the gk99 and tm764 alleles. (B) Representative images of the mitotic regions of wild-type, wrn-1(gk99), and wrn-1(tm764) gonads from one-day-old adult worms immunostained with WRN-1 antibody before and after hydroxyurea (HU, 25 mM) treatment for 8 h. In the nuclei of wrn-1(tm764) gonads, WRN-1 is significantly increased by HU treatment, but not to the same level as in wild-type gonads. (C) Morphological changes of DAPI-stained nuclei in mitotic germ cells after HU treatment. After HU treatment, the premeiotic nuclei in wild-type and wrm-1(tm764) gonads were substantially enlarged and reduced in number. In contrast, much smaller nuclei, some of which were condensed, were observed in the wrn-1(gk99) and wrn-1(RNAi) gonads. Magnification bars are (B) 10 µm and (C) 25 µm. (D) L1-stage worms were irradiated with γ-rays (20 Gy) and their growth was measured after 48 h at 20°C. (E) Developmental abnormalities were scored 3 days after IR (60 Gy) at the L1 stage. Small, small body; Ruptured, ruptured body; Transp, transparent; Unc, uncoordinated movement. (F) Hatching rates were measured for F1 embryos collected 0–24, 24–48, and 48–72 h periods after exposing L4 stage worms of the P0 generation to IR (75 Gy). (0.33 MB PDF) [file pgen.1000801.s001.pdf]

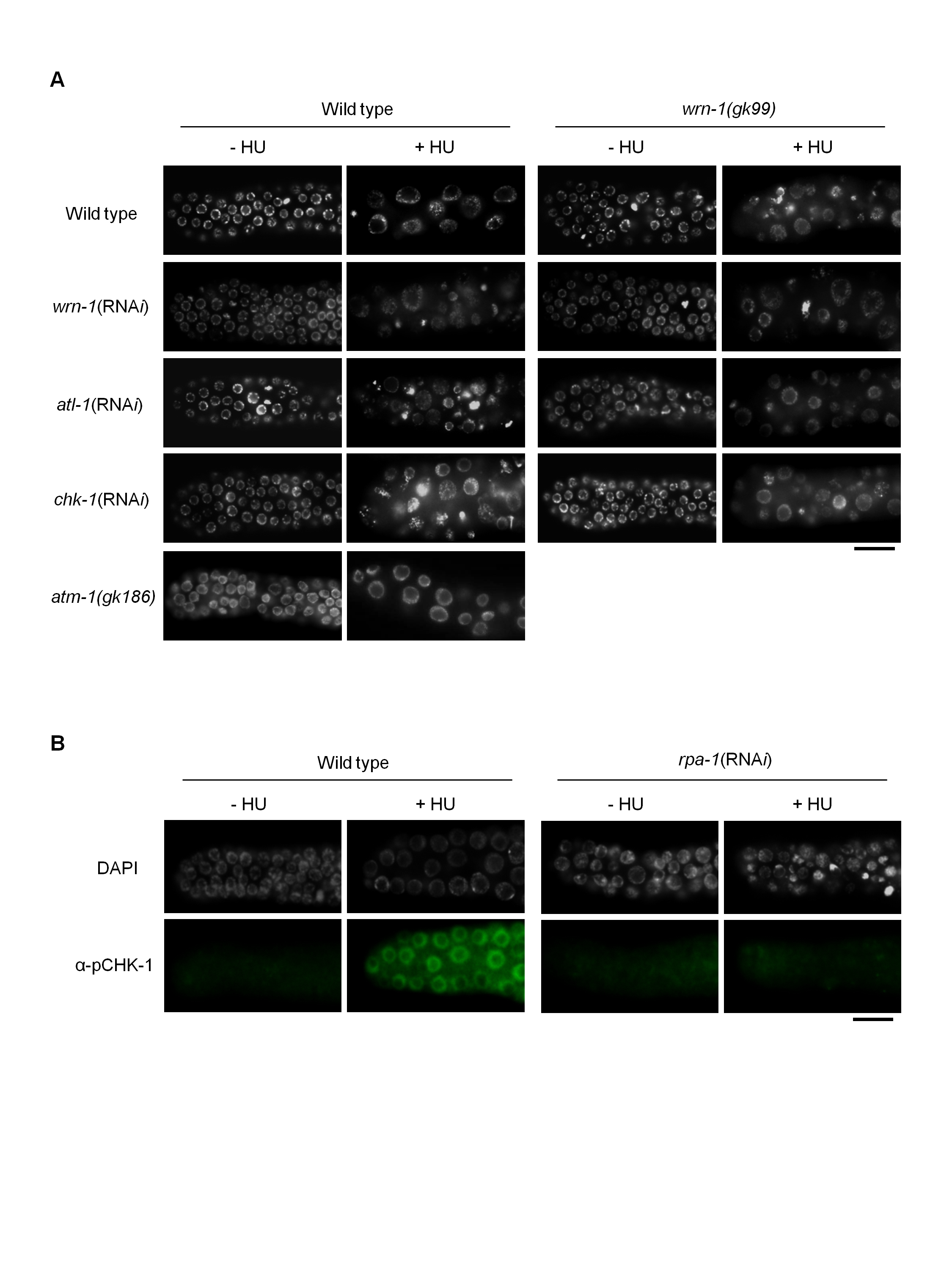

Supplement: Figure S2 — Epistatic relationships of WRN-1 with checkpoint proteins in the cell cycle arrest induced by hydroxyurea. (A) Knockdown of wrn-1, atl-1, and chk-1 were performed from the L1 stage. Images are DAPI-stained nuclei in the gonads of worms, untreated (−HU) or exposed to 25 mM hydroxyurea (+HU) from the L4 stage for 16 h. After HU treatment, premeiotic nuclei in wild-type and atm-1(gk186) gonads were substantially enlarged and reduced in number. In contrast, much smaller nuclei, some of which were condensed, were observed in the mitotic regions of wrn-1(RNAi), atl-1(RNAi), and chk-1(RNAi) gonads. Double deficiencies of atl-1 or chk-1 in the wrn-1(gk99) background did not increase the nuclear phenotype, compared with the single deficiencies of atl-1, chk-1, and wrn-1. wrn-1(RNAi) in the background of wrn-1(gk99) did not change the nuclear phenotype, supporting that wrn-1(gk99) is a null mutation. (B) Knockdown of rpa-1 was carried out from the L4 stage for 16 h before HU treatment, and premeiotic germ cells were probed with phospho-CHK1(S345) antibody. rpa-1 knockdown induced the nuclear phenotype as for the knockdown of atl-1 or chk-1, and abolished phosphorylation of CHK-1(S345). Magnification bars are 25 µm. (1.23 MB TIF) [file pgen.1000801.s002.tif]

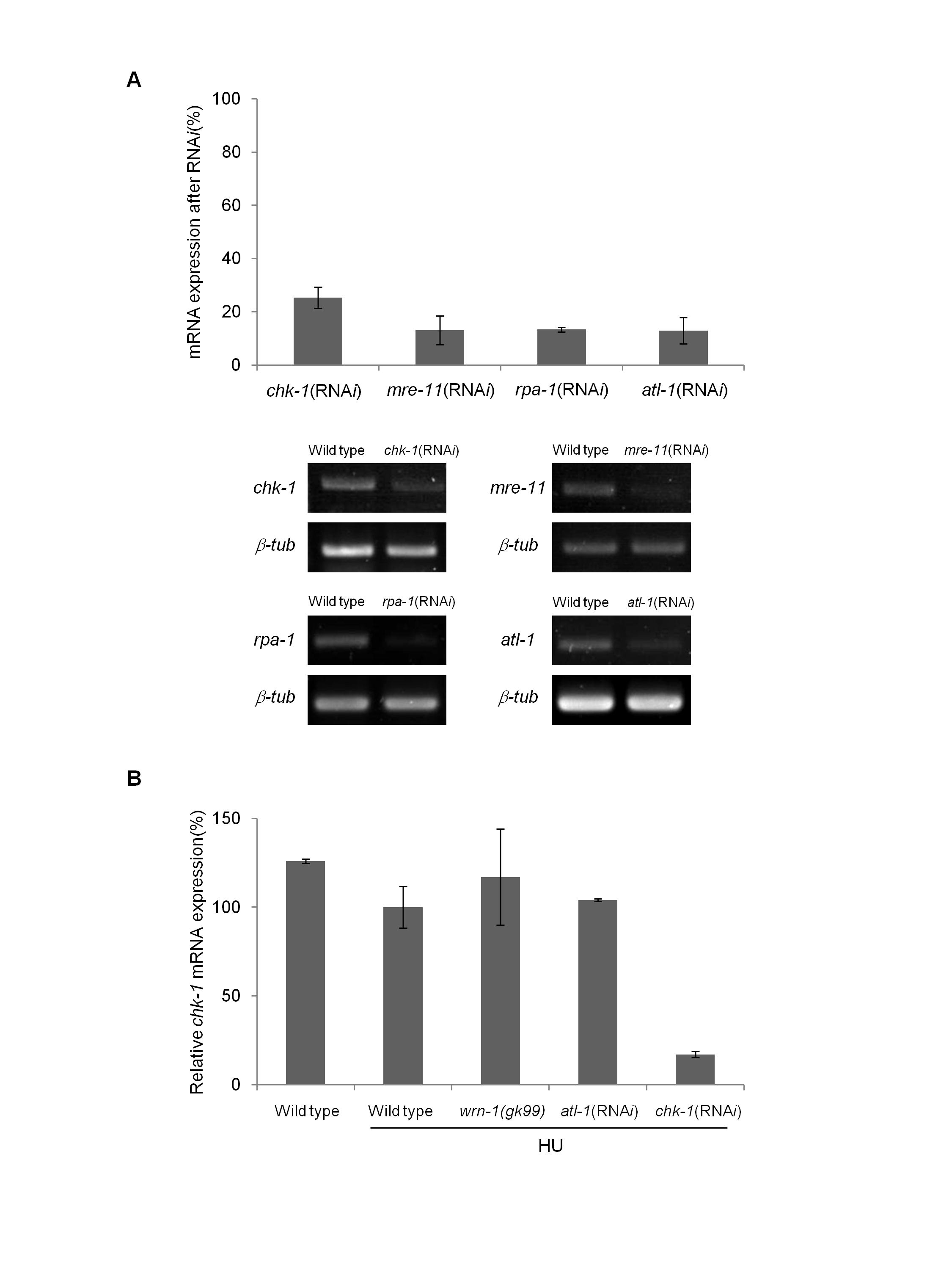

Supplement: Figure S3 — Efficient knockdown of target mRNA expressions and no significant effects of other deficiencies on the chk-1 mRNA expression. Total RNA was isolated form worms after performing feeding RNAi for 16 h. Reverse transcription after random priming and real-time PCR using gene-specific primers were followed. (A) The mRNA levels of target genes (chk-1, mre-11, rpa-1, and atl-1) were estimated by analyzing cDNA amplication kinetics, normalized to that of β-tubulin, and plotted as percent ratios of the corresponding value of the wild-type. The error bars indicate standard errors of the mean. The amplified cDNA fragments were separated on 0.7% agarose gels after 25 PCR cycles. (B) No significant effects of wrn-1 or atl-1 deficiency on the mRNA expression of chk-1 (p values of t test>0.5). The chk-1 mRNA levels were estimated as in (A) before and after HU treatment. (0.32 MB TIF) [file pgen.1000801.s003.tif]

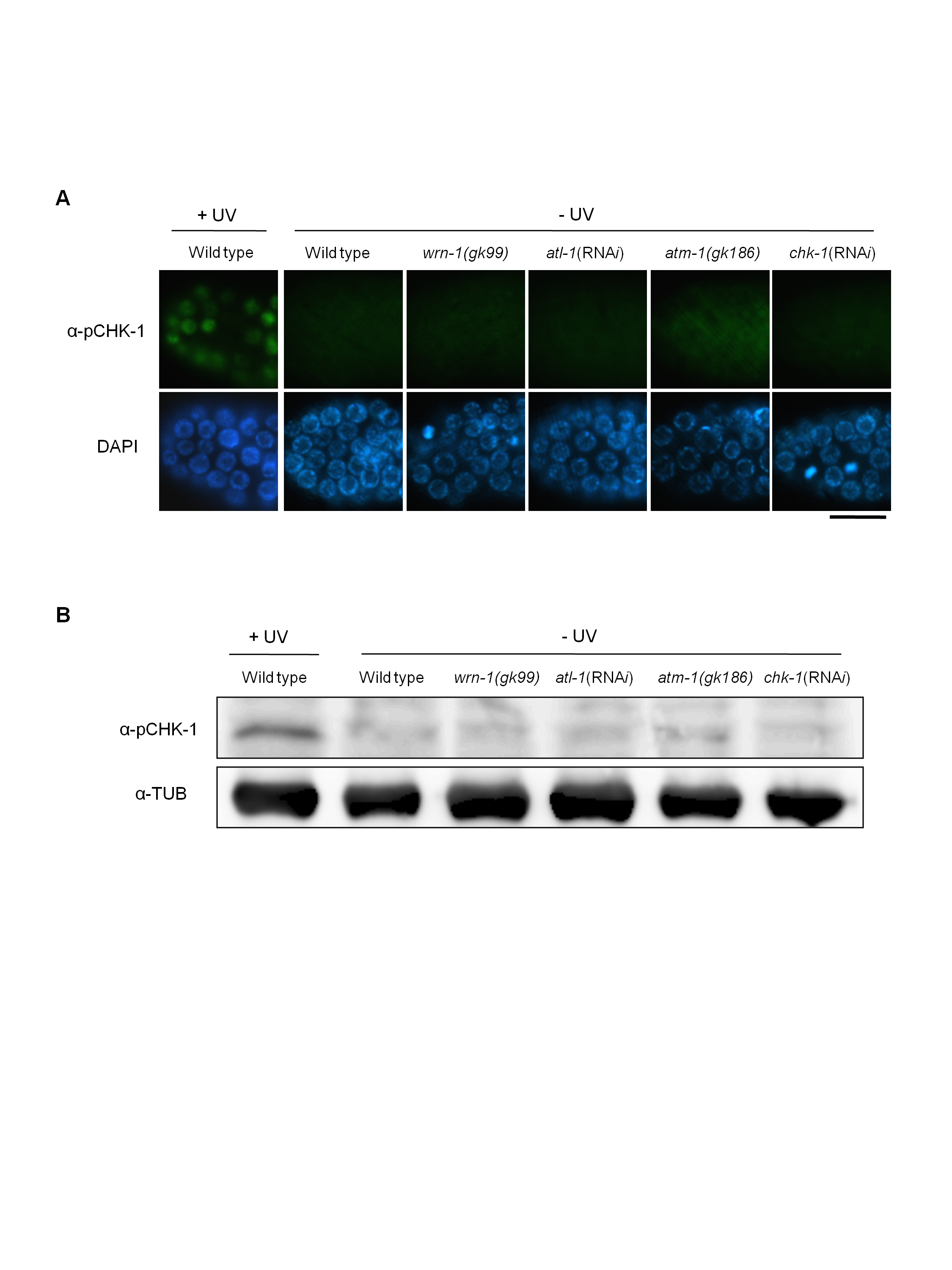

Supplement: Figure S4 — Absence of CHK-1(S345) phosphorylation in untreated germ cells deficient in WRN-1 or checkpoint proteins. (A) Phosphorylation of CHK-1(S345) in mitotic germ cells probed with phospho-CHK1(S345) antibody. Magnification bar, 10 µm. (B) Worm extracts analyzed by western blotting using antibodies to phospho-CHK1(S345), and α-tubulin as a control. (0.97 MB TIF) [file pgen.1000801.s004.tif]

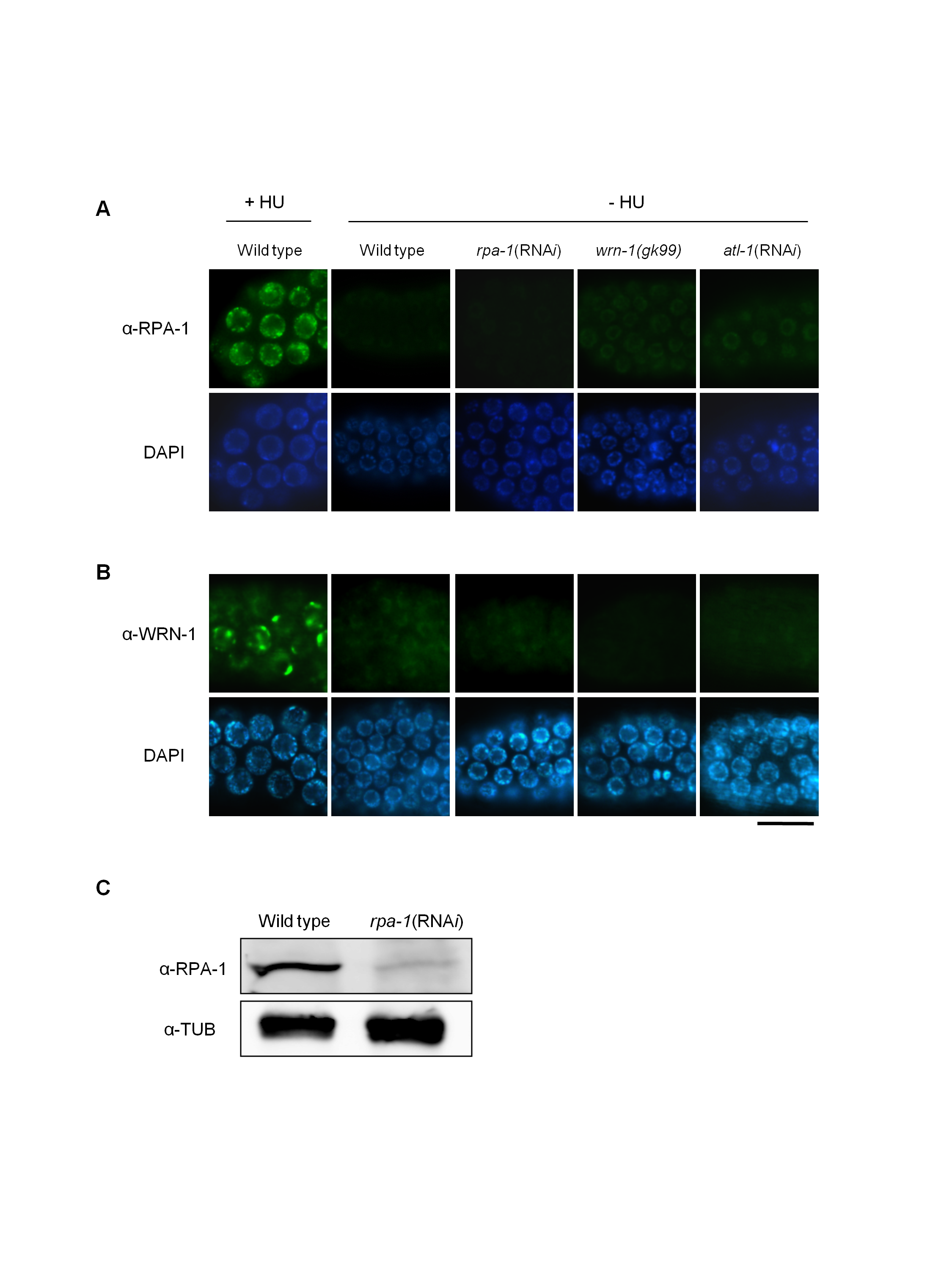

Supplement: Figure S5 — Absence of RPA-1 and WRN-1 focus formation in untreated germ cells, and specificity of RPA-1 antibody. Absence of (A) RPA-1 focus formation and (B) WRN-1 spot formation in germ lines deficient in rpa-1, wrn-1, or atl-1 before hydroxyurea (HU) treatment. (C) Western analysis of RPA-1 in worm extracts after the knockdown, and of α-tubulin as a control. Magnification bar, 10 µm. (1.34 MB TIF) [file pgen.1000801.s005.tif]

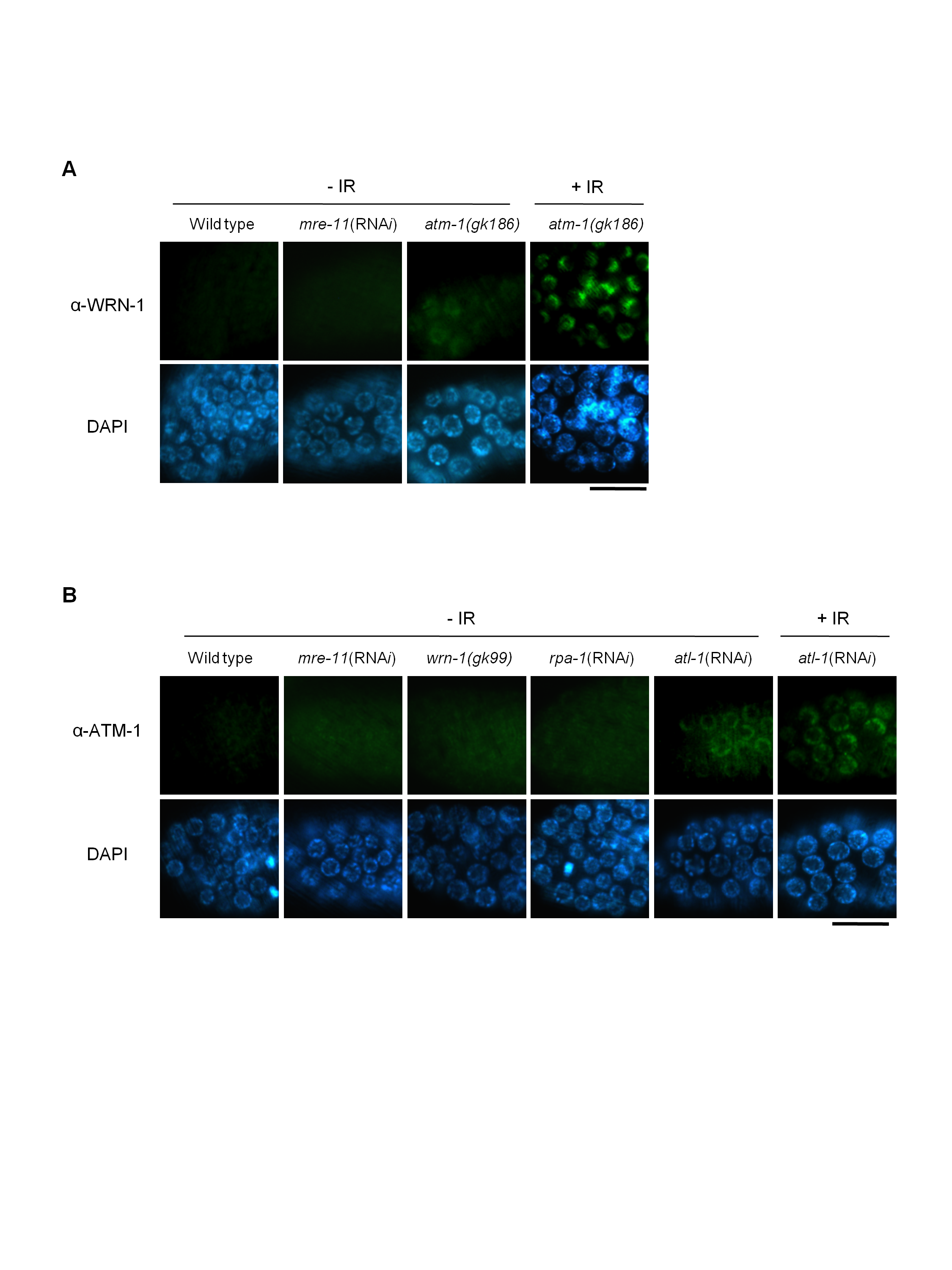

Supplement: Figure S6 — No significant effects of checkpoint proteins on the nuclear localization of WRN-1 and ATM-1 in untreated gonads. (A) Lack of significant effects of mre-11 or atm-1 deficiency on the nuclear localization of WRN-1, and (B) of mre-11, wrn-1, or rpa-1 deficiency on the nuclear localization of ATM-1 in untreated gonads. However, atl-1 deficiency slightly induced the nuclear localization of ATM-1 in untreated gonads, but the level reached was much lower than after IR. Worms were irradiated as one-day-old adults with IR (75 Gy) and cultured for 1 h before immunostaining. Magnification bars, 10 µm. (1.43 MB TIF) [file pgen.1000801.s006.tif]
